# Risk for Asthma in Offspring of Asthmatic Mothers versus Fathers: A Meta-Analysis

**DOI:** 10.1371/journal.pone.0010134

**Published:** 2010-04-12

**Authors:** Robert H. Lim, Lester Kobzik, Morten Dahl

**Affiliations:** 1 Department of Environmental Health, Harvard School of Public Health, Boston, Massachusetts, United States of America; 2 Department of Clinical Biochemistry, Copenhagen University Hospital Herlev, Copenhagen, Denmark; 3 Department of Pulmonary Medicine, Children's Hospital Boston, Boston, Massachusetts, United States of America; 4 Department of Pathology, Brigham and Women's Hospital, Boston, Massachusetts, United States of America; UCL Institute of Child Health, United Kingdom

## Abstract

**Background:**

Many human epidemiologic studies demonstrate that maternal asthma confers greater risk of asthma to offspring than does paternal disease. However, a handful have shown the opposite. Given this disparity, a meta-analysis is necessary to determine the veracity and magnitude of the “maternal effect.”

**Methodology/Principal Findings:**

We screened the medical literature from 1966 to 2009 and performed a meta-analysis to compare the effect of maternal asthma vs. paternal asthma on offspring asthma susceptibility. Aggregating data from 33 studies, the odds ratio for asthma in children of asthmatic mothers compared with non-asthmatic mothers was significantly increased at 3.04 (95% confidence interval: 2.59–3.56). The corresponding odds ratio for asthma in children of asthmatic fathers was increased at 2.44 (2.14–2.79). When comparing the odds ratios, maternal asthma conferred greater risk of disease than did paternal asthma (3.04 vs. 2.44, p = 0.037). When analyzing the studies in which asthma was diagnosed by a physician the odds ratios were attenuated and no significant differences were observed (2.85 vs. 2.48, N = 18, p = 0.37). Similarly, no significant differences were observed between maternal and paternal odds ratios when analyzing the studies in which the patient population was 5 years or older (3.15 vs. 2.60, p = 0.14). However, in all cases the trend remained the same, that maternal asthma was a greater risk factor for asthma than paternal.

**Conclusions/Significance:**

The results show that maternal asthma increases offspring disease risk to a greater extent than paternal disease.

## Introduction

Asthma is a major public health issue. In the United States alone, it affects 6.2 million children and 13.8 million adults, and is a significant cause of morbidity and mortality [1]. Globally, it affects an estimated 300 million people, and is responsible for approximately 1 out of every 250 deaths[2]. Especially troubling is that it has increased significantly in the past 2–3 decades in the U.S. and worldwide [3], [4]. Reasons for this increase are not clear, however may reflect increased exposure to environmental risk factors.

Human epidemiologic studies have attempted to elucidate risk factors for disease. Many, but not all, of these studies have supported the common clinical perception that maternal asthma is associated with increased asthma risk in offspring, as compared to paternal asthma. These studies have varied in design, population composition, asthma definitions and size. Although most experts would agree that maternal asthma is a risk factor for offspring asthma, because of the differences in study design, it is difficult to determine if the magnitude of this ‘maternal effect’ is greater than the ‘paternal effect.’ Therefore, we screened the medical literature and performed a meta-analysis to examine the effect of maternal asthma on offspring asthma susceptibility.

Determination of the magnitude of this effect is important, as it may have broad implications in asthma pathogenesis. If maternal asthma does confer greater asthma risk to offspring than do paternal or parental asthma, then it implies that *in utero* and/or post-natal non-genetic factors can contribute to asthma susceptibility. If *in utero* and/or post-natal exposures can affect asthma initiation, then prevention of asthma becomes a possibility. This also opens new avenues for scientific investigation into the mechanisms underlying asthma susceptibility.

## Methods

### Ethics

No ethical approval was required.

### Searching

**Figure 1:**
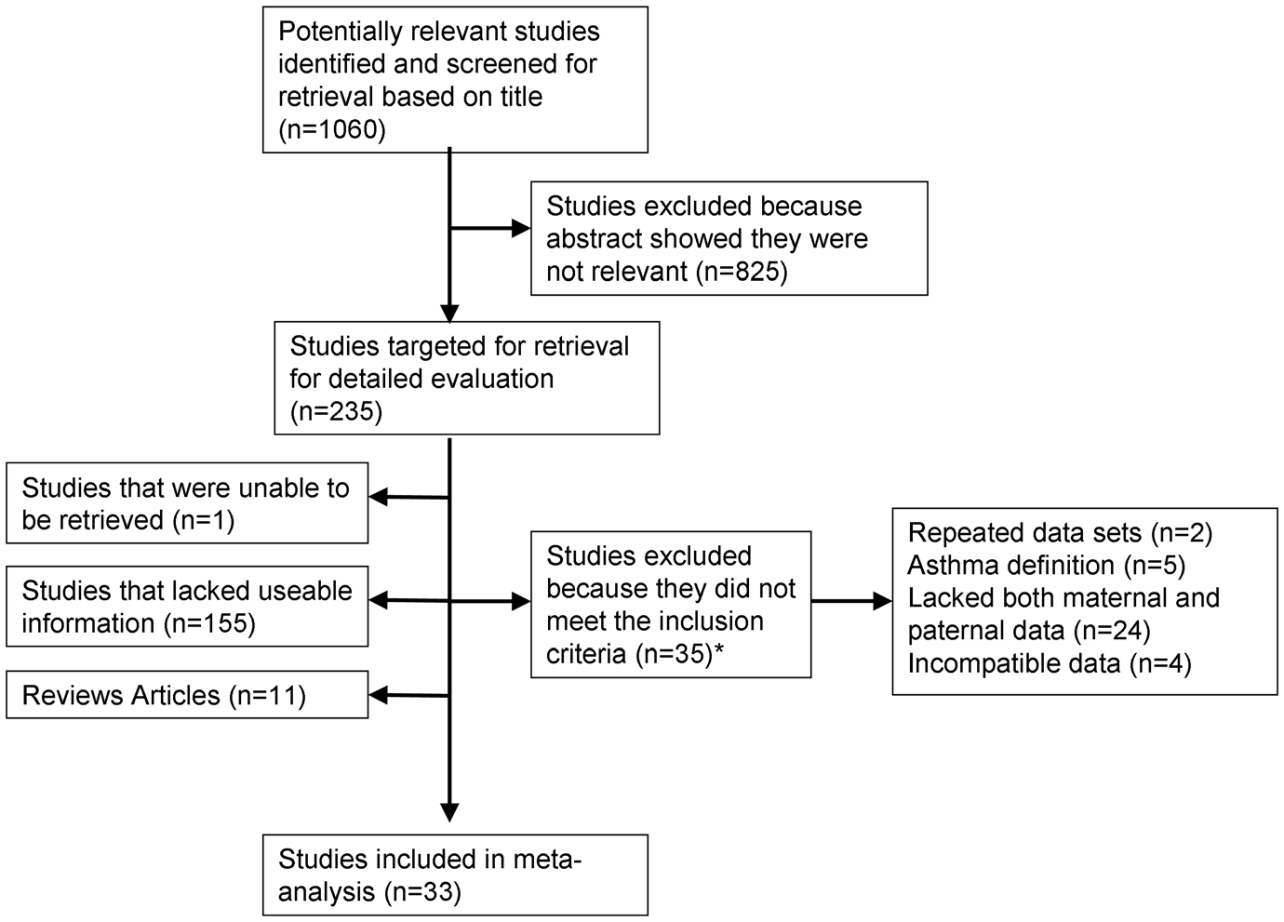
Flow diagram of study selection for the meta-analyses. *see text for details of excluded studies.

A search of the PubMed database was performed in an attempt to identify all studies that examined maternal and paternal asthma as a risk factor for offspring asthma. Records were searched from January 1966 to September 2009. Search headings used were ‘asthma’ and ‘epidemiology’. The titles and abstracts of the articles were then manually scanned to determine relevant studies with information on risk for asthma in offspring of mothers and fathers with asthma. These articles were then retrieved (Figure 1). Bibliographies of pertinent articles and reviews were searched for additional references. The meta-analysis was performed according to the PRISMA guidelines (Table S1, Figure S1).

### Selection

Inclusion criteria: We included case-control and cross-sectional studies that examined asthma risk in offspring of asthmatic mothers and fathers. Offspring asthma was defined by a physician's diagnosis or questionnaire (self-reported asthma, recurrent wheeze, asthma symptoms). Exclusion criteria: 1) Repeated data sets. In a case where different studies used the same database, the more current study was used. 2) Diagnosis of asthma at <1 year of age only. 3) Studies that defined asthma simply as any episode of wheeze. 4) Studies that did not contain both maternal and paternal asthma data. 5) Data that was not compatible with meta-analysis (prevalence rates, relative risks, and regression). We used odds ratios and if they were not available or derivable from the paper, the study was excluded.

### Validity Assessment

The study quality was assessed using the following questions. 1) Was asthma diagnosed by a physician? 2) Were the patients in the study >/ = 5 years old? 3) Was the study a population based or case-control study? 4) Did the study control for ethnicity?

Studies using physician diagnosis as their definition for asthma were considered higher quality studies. Studies using other definitions (i.e. self-reported physician diagnosed asthma, self-reported asthma, self-reported recurrent/persistent wheeze, and self-reported recurrent asthma symptoms; or a combination of these measures) were deemed lower quality. If a study used a combination of definitions (i.e. physician diagnosed asthma or asthma symptoms), the quality of the study was based on the lesser definition. Studies where patients were >/ = 5 years old were considered higher quality as compared to those where patients were <5 years old. This quality measure was used as not all who wheeze as infants/toddlers go on to be diagnosed with asthma. An older patient population implies that the asthma diagnosis is more accurate. Population based studies were deemed higher quality than case-control studies. Studies which controlled for ethnicity were deemed higher quality than those that did not.

### Data Abstraction

Two investigators independently searched and evaluated studies for inclusion (RHL, MD). Resulting lists were compared. Disagreements were resolved by discussions between RHL and MD. When discussions did not resolve disagreements, a third author (LK) was included.

### Study Characteristics

Table 1 summarizes the characteristics of the studies included in the analysis. Heterogeneity among the studies was assessed using the Q-statistic. Included studies were case-control and cohort studies using the categorical outcome of asthma (physician diagnosis, self report of physician diagnosis, or self report) and recurrent wheeze.

**Table 1:** Studies examining risk of asthma in offspring from asthmatic mothers and fathers.

				Offspring		Adjusted risk estimate
Author (ref #)	Year	Country	N	Asthma definition	Age range, yrs	
Dold (8)	1992	Germany	4447	Quest MD asthma or recurrent wheeze	9 to 11	None
Frischer (12)	1993	Germany	1812	Quest MD asthma	7 to 8	Yes
Kelly (16)	1995	UK	3746	Quest MD asthma	5 to 11	Yes
Abramson (36)	1996	Australia	675	Current MD ashtma	20 to 44	None
Ehrlich (10)	1996	S.Africa	620	Quest current asthma/wheeze	7 to 9	None
BaezaBacab (37)	1997	Mexico	505	Quest MD asthma	6 to 12	None
Rona (28)	1997	UK, Scotland	11924	Quest asthma attack or persistent wheeze	5 to 11	Yes
Jenkins (23)	1997	Australia	26718	Quest asthma attack or wheezy breathing	0 to 32	None
Litonjua (17)	1998	USA	740	Quest MD asthma	1 to 24	Yes
Millar (38)	1998	Canada	22433	Quest asthma	0 to 11	Yes
Halonen (33)	1999	USA	1246	Quest MD asthma	6	None
Illi (21)	2001	Germany	939	Current MD Asthma	7	Yes
Wang (19)	2001	Taiwan	414	Quest MD asthma and symptoms	11 to 16	Yes
Sherriff (30)	2001	UK	1536	Quest persistent wheeze	3.5	Yes
Wickens (20)	2001	New Zealand	474	Quest MD asthma and medicines	7 to 9	Yes
Karunasekera (24)	2001	Sri Lanka	600	MD asthma	1 to 10	Yes
Jaakkola (22)	2001	Norway	2531	Quest MD asthma and symptoms	4	Yes
El-Sharif (35)	2003	Palestine	351	MD asthma	6 to 12	None
Cole Johnson (32)	2004	USA	222	Current MD asthma	6 to 7	Yes
Sandin (29)	2004	Sweden	719	Quest persistent wheeze	4	Yes
Jan (14)	2004	Taiwan	2076	Quest MD asthma or symptoms	18 to >65	Yes
Arshad (34)	2005	UK	1373	Quest asthma and wheeze	10	Yes
Taveras (31)	2006	USA	1101	Quest persistent wheeze	2	Yes
De Sario (7)	2006	Italy	1674	Quest persistent wheeze	9 to 11	Yes
Lee (25)	2006	Taiwan	24784	Quest asthma	26 to 50	Yes
Elizur (11)	2007	Israel	91	Quest asthma and lung function tests	6 to 40	None
Bjerg (6)	2007	Sweden	3430	Current MD asthma	7 to 8	Yes
Mai (26)	2007	Canada	723	MD asthma	8 to 10	None
Morais-Almeida (18)	2007	Portugal	249	Recurrent wheeze	8 to 14	Yes
Martel (27)	2008	Canada	109746	MD asthma and medicines	10	Yes
Jacobson (13)	2008	USA	517	Quest MD asthma and medicines	4 (mean)	Yes
Dong (9)	2008	China	16789	Quest MD asthma	2 to 13	Yes
Karino (15)	2008	Japan	9615	Quest asthma	18 to 20	Yes

In the Jan study asthma included probable asthmatics. In the Sheriff and Martel studies maternal asthma was asthma during pregnancy, while paternal asthma was defined by medical history. In the Johnson study parental asthma included hay fever and allergies. Medical doctor (MD) asthma  =  physician-diagnosed asthma. Quest MD asthma  =  physician-diagnosed asthma on questionnaire or by parents report.

### Data Analysis

Meta-analyses were performed using the MIX program version 1.7 [5]. Data from studies with dichotomous asthma outcomes were combined to give a summary odds ratio using the inverse variance method. Depending on the test for heterogeneity among the studies, we used a fixed-effects model or a random-effect model for the meta-analysis. Funnel plots and Egger's regression test were used to search for publication bias. Subgroup analyses were used to examine three potentially important sources of heterogeneity. The predetermined subgroups were based on asthma definition, the age of offspring, and study design. The stability of the summary risk estimate was evaluated using a sensitivity analysis in which each study was individually removed and the odds ratio was recalculated. Removal of each individual study did not significantly alter the summary odds ratio for offspring asthma in maternal asthma (range of recalculated summary odds ratio: 2.82 to 3.12) or paternal asthma (range of recalculated summary odds ratios: 2.36 to 2.53). The summary odds ratios for offspring asthma in maternal and paternal asthma were compared as described [6].

## Results

The initial Pubmed search using search headings ‘asthma’ and ‘epidemiology’ resulted in approximately 15,000 journal articles. Based on the titles 1,060 were potentially relevant, of which 235 were targeted for retrieval after review of the abstracts (Figure 1). One study was unavailable for retrieval [7], thus 234 total articles were subjected to detailed review. The 33 studies [8]–[40] included in the meta-analysis are listed in Table 1.

Of the 234 papers retrieved for detailed evaluation, a total of 201 were excluded. 155 were excluded for lack of useable information. 11 were excluded because they were reviews [41]–[51]. An additional 35 studies were excluded for reasons summarized in figure 1 [52]–[85]. The 4 studies that were removed due to ‘incompatible data’ did not use odds ratios, nor were they derivable from the data presented [82]–[85]. The remaining 33 were used for meta-analysis.

### Study Quality

Table 1 lists the characteristics of the 33 papers. In 18 studies, asthma was physician diagnosed based on questionnaire [8], [11], [14], [15], [18], [19], [21]–[24], [26], [28], [29], [34], [35], [37]–[39]. In 7 studies asthma diagnosis was based on questionnaire, but not specifically physician diagnosed [12], [13], [16], [17], [25], [27], [40]. In 6 studies, recurrent/persistent wheeze was used as a surrogate for asthma [9], [10], [20], [30]–[32]. Of the 7 studies with non-physician diagnosed asthma, 1 included recurrent wheeze [30] in its asthma definition, and 1 included “wheezy” breathing [25]. Of the 33 studies used in this meta-analysis, 8 included patients less than 5 years of age at time of the study [11], [15], [19], [20], [26], [32], [33], [40]. Of the 33 studies used in the meta-analysis, 25 were population based [8]–[11], [13]–[20], [23]–[25], [27], [30]–[35], [39], [40], [86] and 8 were case-control studies [12], [21], [22], [26], [28], [29], [37], [38].

Although asthma epidemiology can vary based on ethnicity[1], [87]–[90], there is no direct evidence that ethnicity confounds a potential relationship between maternal/paternal and offspring asthma. However, ethnicity could confound our data, if couples were unbalanced on this convariate. Of the 33 studies, 9 were conducted in the North America, 8 in Europe, 1 in Africa, 2 in Australia, 1 in New Zealand, 6 in Asia, 4 in the U.K., and 2 in the Middle East (Table 1). Ethnic diversity was presumed to be less in populations from countries other than North America and the U.K.

Of the studies used in this meta-analysis, only 3 fulfilled all 4 predetermined quality criteria [8], [14], [23]. The most common reason was lack of physician diagnosis.

### Maternal asthma and offspring asthma

**Figure 2:**
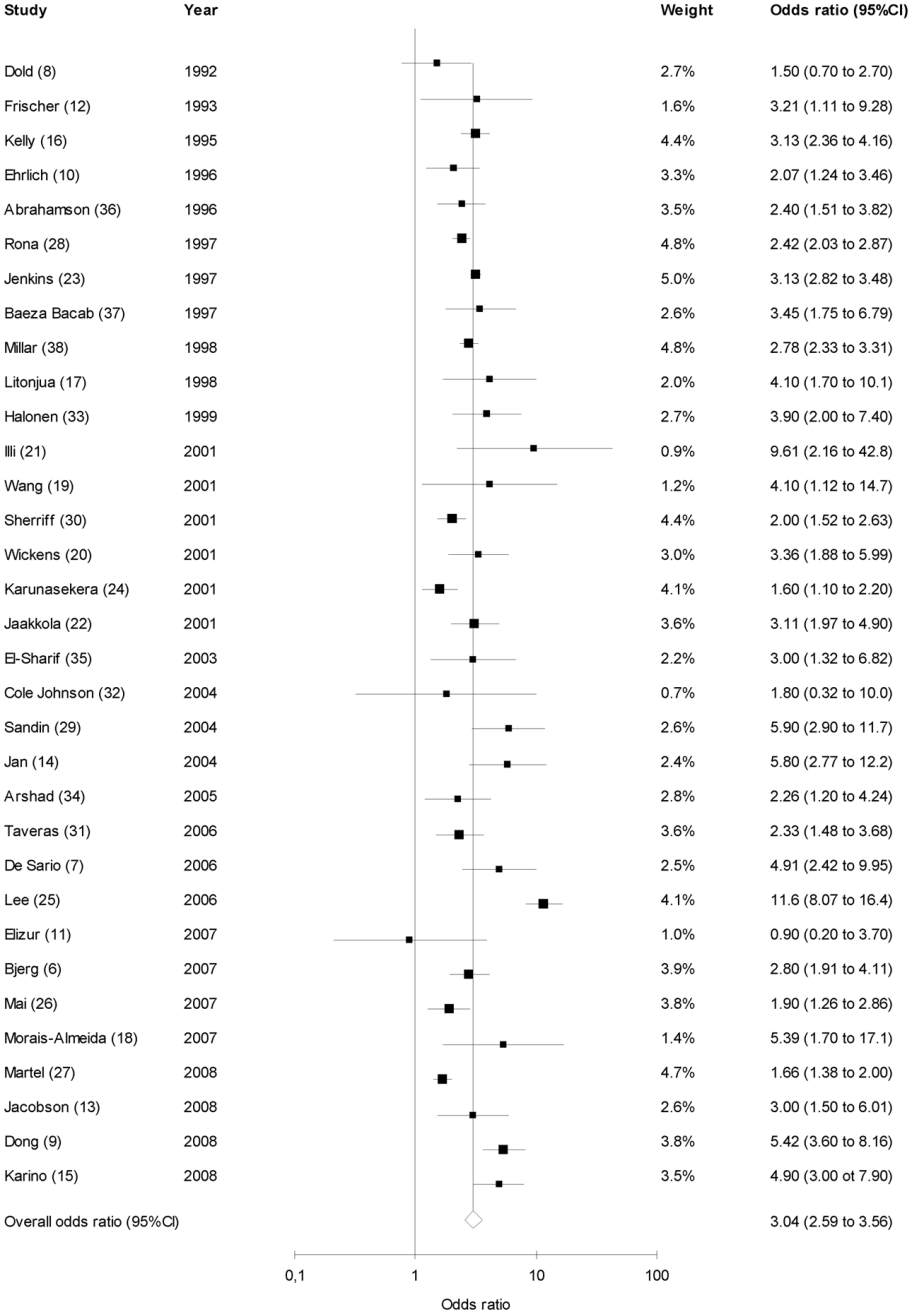
Studies of maternal asthma as a risk factor of asthma. Sizes of boxes represent inverse variance weights (random effects model). Lines represent 95% confidence intervals.

The pooled analysis for the 33 total studies is summarized in figure 2. Children of asthmatic mothers are more likely than children of non-asthmatic mothers to develop asthma (summary OR 3.04, 95% CI: 2.59–3.56). The test for heterogeneity showed a non-significant p-value of 0.13, indicating that the studies had similar outcomes and were appropriate to be summarized in a meta-analysis. The funnel plot of OR versus standard error was symmetric (Figure S2), and Egger's test was negative for publication bias (p = 0.38). To ensure that no single study skewed the overall results, each study was removed one at a time and the summary OR recalculated. Removal of each individual study did not significantly alter the summary OR. All the recalculated summary odds ratios lay within the 95% confidence interval of the principal analysis (range of recalculated summary odds ratio: 2.82 to 3.12).

Subgroup analysis was also performed based on quality measures. When analyzing only those papers which were based on physician diagnosed asthma (including self-report physician diagnosed asthma) (N = 18) similar but attenuated results were seen 2.85 (2.30–3.54). When analyzing those studies where the patient population was >/ = 5 year of age (N = 25) or those which were population based (N = 25) the summary OR was similar but elevated at 3.15 (2.53–3.93) and 3.41 (2.87–4.06) respectively.

We also analyzed only those studies that adjusted for potential confounding and those studies where the patient population were adults (age >/ = 18 years). When analyzing those studies that adjusted for potential confounding (N = 24) or those where the patient population was >/ = 18 year of age (N = 4) similar but elevated summary OR were seen (3.34 (2.69–4.14) and 5.33 (2.51–11.3) respectively).

### Paternal asthma and offspring asthma

**Figure 3:**
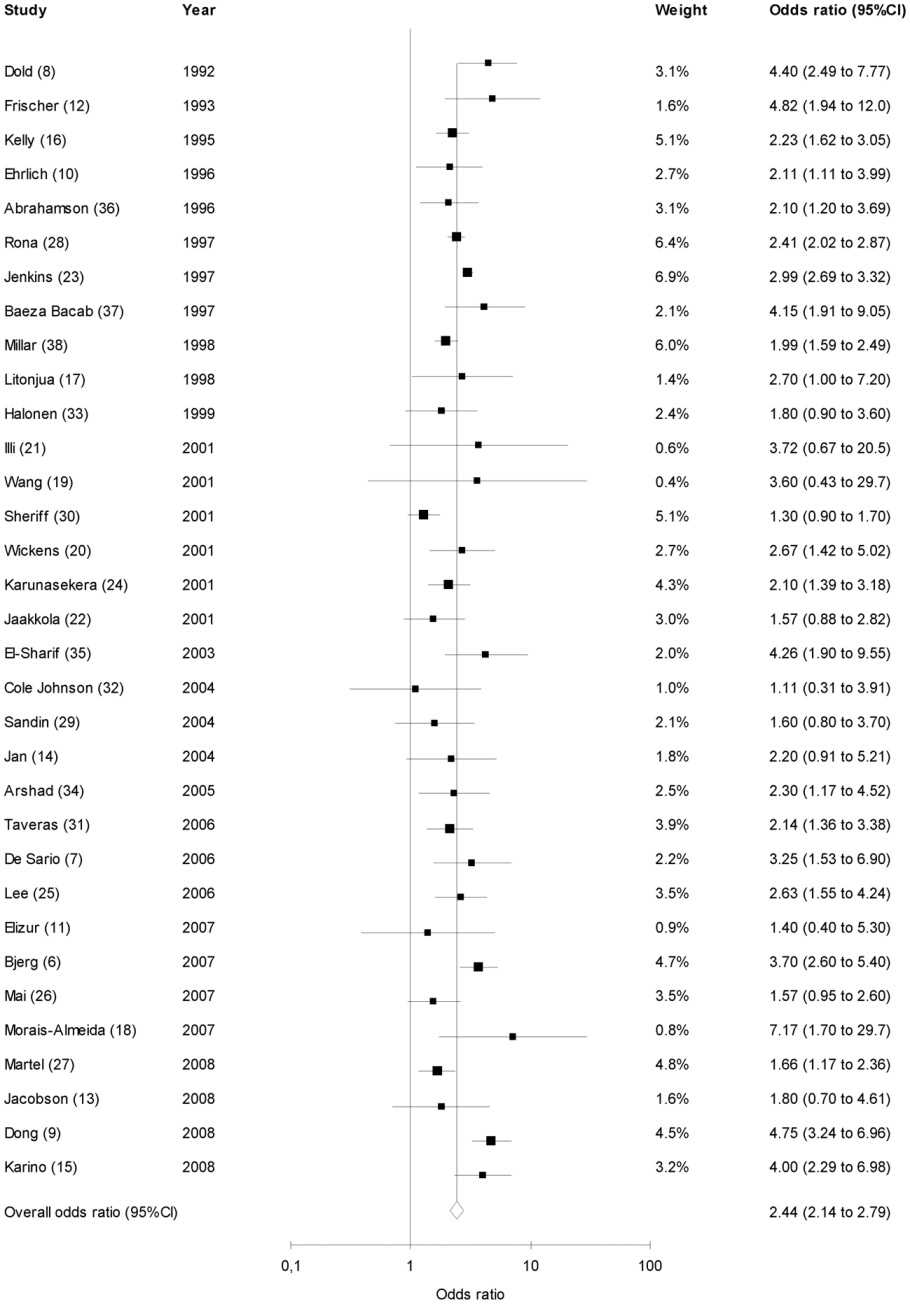
Studies of paternal asthma as a risk factor of asthma. Sizes of boxes represent inverse variance weights (random effects model). Lines represent 95% confidence intervals.

Similar analysis was performed on the paternal asthma data. The analysis is summarized in figure 3. Children of asthmatic fathers are more likely to develop asthma than those of non-asthmatic fathers (summary OR 2.44, 95%CI: 2.14–2.79). The test for heterogeneity showed a non-significant p-value of 0.06. The funnel plot of OR versus standard error was symmetric (Figure S3), and Egger's test was negative for publication bias (p = 0.50). To ensure that no single study skewed the overall results, each study was removed one at a time and summary OR recalculated. Removal of each individual study did not significantly alter the summary OR (range of recalculated summary odds ratios: 2.36 to 2.53).

Subgroup analysis was also performed in the same groups as were used in the maternal asthma data. When analyzing only those papers which were based on physician diagnosed asthma (N = 18) similar results were seen 2.48 (2.01–3.06). When analyzing those studies where the patient population was >/ = 5 year of age (N = 25) or those which were population based (N = 25) the summary OR was similar but elevated at 2.60 (2.28–2.96) and 2.56 (2.19–2.98) respectively; Heterogeneity was possible in the former subgroup analysis as p = 0.02.

When analyzing those studies that adjusted for potential confounding (N = 24) or those where the patient population was >/ = 18 year of age (N = 4) similar but elevated summary odds ratio were seen (2.39 (2.04–2.80) and 2.72 (2.03–3.65) respectively).

### Maternal versus Paternal Effect

The data show that both maternal and paternal disease state affects offspring disease, and based on individual studies, maternal asthma is the more potent contributor. When analyzing all studies and comparing the two summary odds ratios, maternal asthma confers greater risk of disease than does paternal asthma (OR 3.04 and 2.44, respectively, p = 0.037). Similar trends were seen using subgroup analysis, although the differences did not achieve statistical significance for all analyses. For example, in analyzing the studies in which the patient population was >/ = 5 yo the odds ratios were 3.15 and 2.60, maternal versus paternal asthma respectively (p = 0.14). Similarly in analysis of the studies in which asthma was diagnosed by a physician the odds ratios were 2.85 and 2.48, maternal versus paternal asthma respectively (p = 0.37). When analyzing the studies which were population based the difference remained, with odds ratios of 3.41 and 2.56, maternal versus paternal respectively (p = 0.02).

We also analyzed those studies which adjusted for potential confounding and those studies where the patient population were adults (>/ = 18 years). Analyzing the studies which adjusted for confounders the odds ratios were 3.34 and 2.39, maternal versus paternal respectively (p = 0.01). When analyzing the studies where the patient population was >/ = 18 years the odds ratios were 5.33 versus 2.72, maternal versus paternal respectively (p = 0.10).

## Discussion

To investigate the role of maternal asthma in offspring asthma, we performed a meta-analysis of multiple studies to determine if a parent of origin effect exists. In our meta-analysis of 33 studies, maternal asthma predisposes offspring to disease more so than paternal asthma. This effect is modest (OR 3.04 versus 2.44), but statistically significant. This demonstrates that non-genetic *in utero* and/or post-natal factors may play a significant role in the transmission of asthma susceptibility. How these factors could induce asthma susceptibility has not been elucidated. However, animal models have demonstrated the potential for transplacental passage of ‘pro-asthmatic’ mediators (e.g. Th2 cytokines, immunologic cells, etc.), which could theoretically be capable of modifying the developing fetal immune system [reviewed [91]]. Alternatively, or in addition to, it is possible that post-natal exposures such as maternal breast milk could shape the developing immune system. Murine models have demonstrated that breast milk from asthmatic mothers can induce asthma susceptibility in offspring [92]. Human studies have also demonstrated that breast feeding can affect offspring asthma/lung function [93], [94]. The mechanisms for the phenomenon demonstrated in this meta-analysis require further study using animal models.

When analyzing the studies in which asthma was diagnosed by a physician, in studies which were population based or in studies with a patient population >/ = 5 years of age, the maternal effect remained more prominent than the paternal effect, though the magnitude of the difference was attenuated for some analyses. The subgroup analyses had fewer individuals and thus reduced power in comparison to the overall analysis. When analysing smaller subgroups subsequent to the principal analysis the chance of spurious findings may increase. Had more studies fit the quality criteria, then the differences may not have been attenuated. There were also potential confounding factors for this analysis that were not considered. These are discussed below.

This meta-analysis has several drawbacks that warrant discussion. There are multiple known risk factors for asthma, which this meta-analysis did not control for. For example, both *in utero* and *ex utero* exposure to tobacco smoke can increase the risk of wheezing/asthma [48], [95], [96]. Lower socioeconomic status is also associated with increased asthma susceptibility [97], [98]. In addition, breasting feeding can affect asthma risk and lung function depending on timing and maternal disease status [93], [94]. This meta-analysis did not exclude studies that did not control for such exposures, nor was it used as quality criteria. This was because few studies controlled for such exposures. Exposure to such factors is likely to happen independent of maternal or paternal disease status. Therefore, inclusion of these studies would make it less likely to discover a significant difference between maternal and paternal asthma. Despite this, the overall summary OR of this study demonstrated that maternal asthma, more so than paternal asthma, is a significant risk factor for offspring asthma. The inclusion of studies that did not control for exposures to asthma risk factors may explain in part why the trend was preserved in subgroup analysis, but lost statistical significance.

It should also be noted that 4 retrieved studies were not used in this analysis because their data was not compatible with our meta-analysis. Of the four papers, two showed a greater role for paternal asthma versus maternal asthma in offspring asthma risk [84], [85]. As these papers used regression, it is difficult to extrapolate how they would have affected the analysis were their data able to be included.

A common problem in meta-analysis is publication bias. Based on Eggers test and a symmetrical funnel plot, this study is free from publication bias. Also, for most of the studies used in this paper, the finding that maternal asthma conferred greater risk to offspring was not the primary endpoint. This further decreased the likelihood of publication bias.

Based on this meta-analysis, maternal asthma increases offspring disease risk to a greater extent than paternal disease. This can be interpreted to mean that the increased asthma risk conferred by maternal disease is not due solely to genetic inheritance. These findings are consistent with experimental studies demonstrating that maternal asthma/exposures in animal models can induce asthma susceptibility in offspring [92], [99]–[101], and support the need for further work in elucidating the mechanisms for the ‘maternal effect.’

## Supporting Information

Table S1 PRISMA Checklist
(0.07 MB DOC)

Figure S1 PRISMA Flowsheet
(0.06 MB DOC)

Figure S2 Funnel plot of studies examining asthma risk in offspring of asthmatic versus non-asthmatic mothers. Individual risk estimates for each study are superimposed on lines representing the summary odds ratio (center) and pseudo 95% confidence limits. There is no evidence of bias in the formal plot or by Eggers test.
(0.03 MB PDF)

Figure S3 Funnel plot of studies examining asthma risk in offspring of asthmatic versus non-asthmatic fathers. Individual risk estimates for each study are superimposed on lines representing the summary odds ratio (center) and pseudo 95% confidence limits. There is no evidence of bias in the formal plot or by Eggers test.
(0.03 MB PPT)
