# Supplementary material for: Risk for Asthma in Offspring of Asthmatic Mothers versus Fathers: A Meta-Analysis
Source: PLoS One. 2010 Apr 12;5(4):e10134. doi: 10.1371/journal.pone.0010134 (PMC2853568; doi:10.1371/journal.pone.0010134)
Supplement: Figure S1 — PRISMA Flowsheet (0.06 MB DOC) [file pone.0010134.s002.doc]

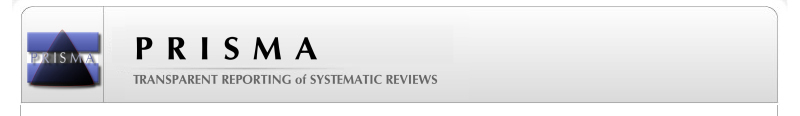
**PRISMA 2009 Flow Diagram**

**Screening**

**Included**

**Eligibility**

**Identification**

Records identified through database searching
(n = 14920 )

Additional records identified through other sources
(n = 0 )

Records after duplicates removed
(n = 14920 )

Records screened by title
(n = 14920 )

Records excluded
(n = 13860)

Full-text articles assessed for eligibility
(n = 235 )

Full-text articles excluded, with reasons:

Studies that were unable to be retrieved (n=1)

Studies that lacked useable information (n=155)

Reviews Articles (n=11)

Repeated data sets (n=2)

Asthma definition (n=5)

Lacked both maternal and paternal data (n=24)

Incompatible data (n=4)

TOTAL N=202

Studies included in qualitative synthesis
(n = 33)

Studies included in quantitative synthesis (meta-analysis)
(n = 33)

Records screened by abstract
(n = 1060 )

Records excluded
(n = 825)
