# Supplementary figures and images for: Risk for Asthma in Offspring of Asthmatic Mothers versus Fathers: A Meta-Analysis
Source: PLoS One. 2010 Apr 12;5(4):e10134. doi: 10.1371/journal.pone.0010134 (PMC2853568; doi:10.1371/journal.pone.0010134)

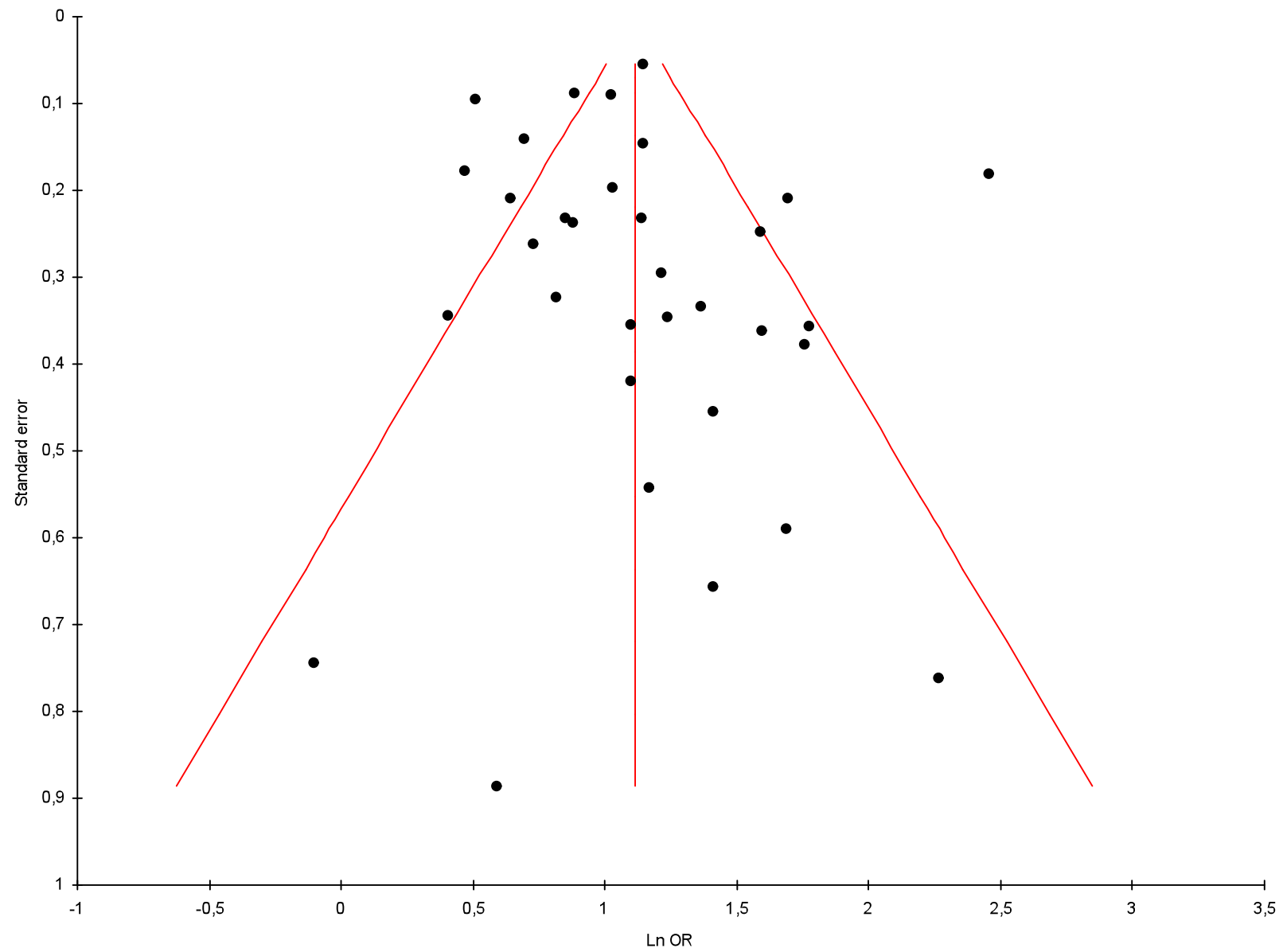

Supplement: Figure S2 — Funnel plot of studies examining asthma risk in offspring of asthmatic versus non-asthmatic mothers. Individual risk estimates for each study are superimposed on lines representing the summary odds ratio (center) and pseudo 95% confidence limits. There is no evidence of bias in the formal plot or by Eggers test. (0.03 MB PDF) [file pone.0010134.s003.pdf]

## Slide 1
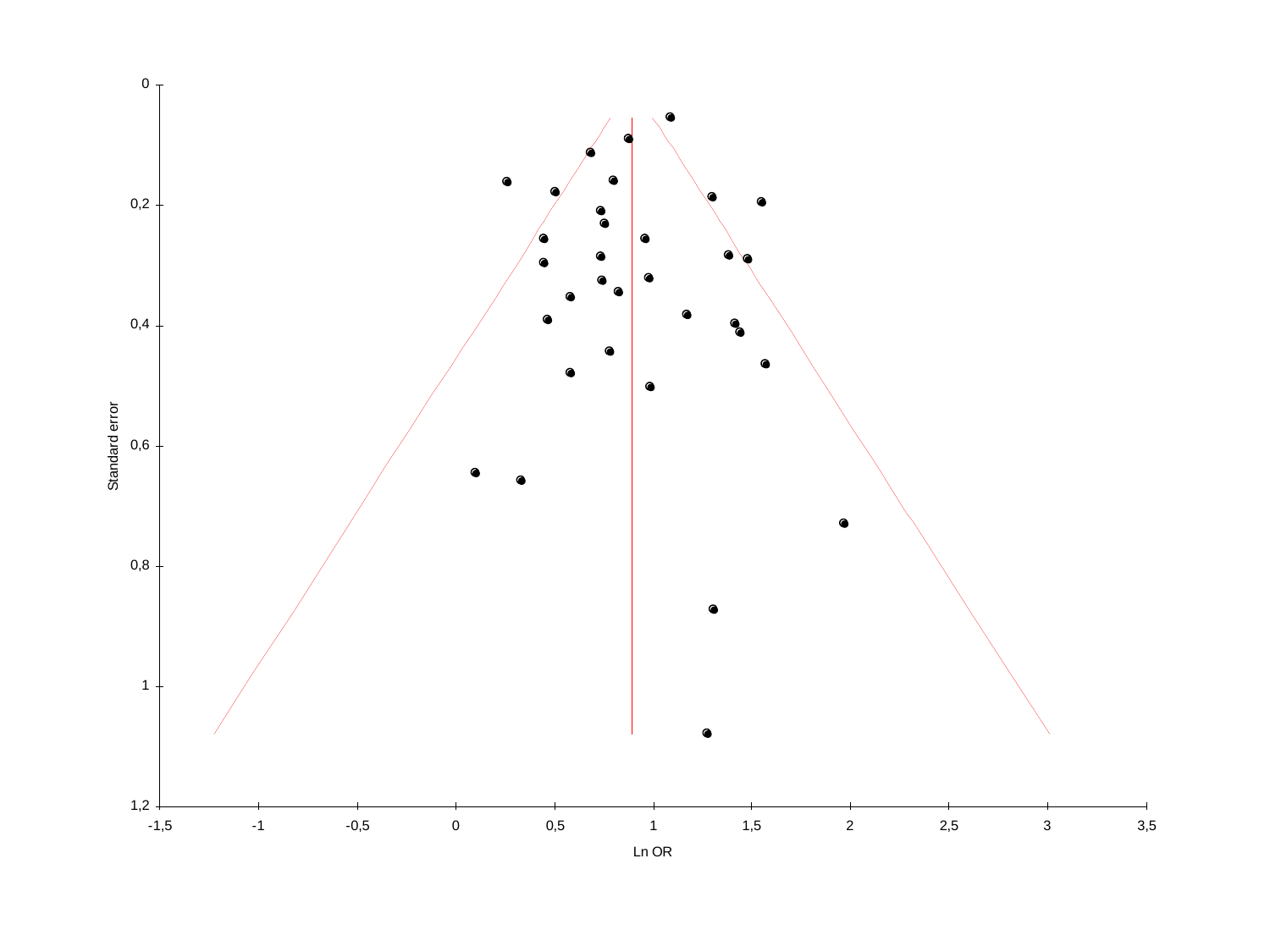

Supplement: Figure S3 — Funnel plot of studies examining asthma risk in offspring of asthmatic versus non-asthmatic fathers. Individual risk estimates for each study are superimposed on lines representing the summary odds ratio (center) and pseudo 95% confidence limits. There is no evidence of bias in the formal plot or by Eggers test. (0.03 MB PPT) [file pone.0010134.s004.ppt]
